# A Learn-to-Rank Approach for Predicting Road Cycling Race Outcomes

**DOI:** 10.3389/fspor.2021.714107

**Published:** 2021-10-06

**Authors:** Leonid Kholkine, Thomas Servotte, Arie-Willem de Leeuw, Tom De Schepper, Peter Hellinckx, Tim Verdonck, Steven Latré

**Affiliations:** ^1^Department of Computer Science, University of Antwerp-IMEC, Antwerp, Belgium; ^2^Department of Mathematics, University of Antwerp, Antwerp, Belgium

**Keywords:** road cycling, machine learning, winner prediction, learn-to-rank, cycling race performance

## Abstract

Professional road cycling is a very competitive sport, and many factors influence the outcome of the race. These factors can be internal (e.g., psychological preparedness, physiological profile of the rider, and the preparedness or fitness of the rider) or external (e.g., the weather or strategy of the team) to the rider, or even completely unpredictable (e.g., crashes or mechanical failure). This variety makes perfectly predicting the outcome of a certain race an impossible task and the sport even more interesting. Nonetheless, before each race, journalists, ex-pro cyclists, websites and cycling fans try to predict the possible top 3, 5, or 10 riders. In this article, we use easily accessible data on road cycling from the past 20 years and the Machine Learning technique Learn-to-Rank (LtR) to predict the top 10 contenders for 1-day road cycling races. We accomplish this by mapping a relevancy weight to the finishing place in the first 10 positions. We assess the performance of this approach on 2018, 2019, and 2021 editions of six spring classic 1-day races. In the end, we compare the output of the framework with a mass fan prediction on the Normalized Discounted Cumulative Gain (NDCG) metric and the number of correct top 10 guesses. We found that our model, on average, has slightly higher performance on both metrics than the mass fan prediction. We also analyze which variables of our model have the most influence on the prediction of each race. This approach can give interesting insights to fans before a race but can also be helpful to sports coaches to predict how a rider might perform compared to other riders outside of the team.

## 1. Introduction

In recent years, the amount of data collected in sports has increased enormously. On the one hand, the usage of sensors on the body (e.g., heart rate monitors) and equipment (e.g., power meter on bicycles) allows detailed profiling of the athlete. On the other hand, the amount of open data recorded by fans and journalists allows for a more fine-grained overview of the team and athlete performance. To turn the abundant data available into insights and knowledge, sports and data scientists make use of Machine Learning (ML) techniques. For example, ML techniques have been applied to assist coaches (Vales-Alonso et al., 2015; Lim et al., 2018), help build teams or scout for new talent (wiklinski et al., 2021), detect events, track players/balls, estimate poses in videos (Thomas et al., 2017), analysis of the physiological determinants of performance, such as the ventilatory thresholds from the cardiopulmonary exercise test (Zignoli et al., 2021), and assess the risk of injury (Claudino et al., 2019).

Another popular application in sports data science is the prediction of sport events outcomes, of which some examples will be presented in the following section. These predictions are beneficial for identifying talent, creating or changing strategies, or purely for entertainment purpose by commentators or fans. Many factors influence the outcomes of a sporting event and as result, predicting those outcomes is a challenging task. For example, in road cycling, the outcome of a race can be influenced by the preparedness of the rider, the fitness of the rider to the course, mental state, and their physical condition as the season progresses. Some factors are external and may even be completely unpredictable, such as strategy of the team, the weather, or crashes during the race.

As so many factors influence the outcomes of a sports competition, they are usually based on high domain knowledge, past observations of the team or athletes, and personal preferences or favorites. As ML techniques have proven themselves useful in cases where domain expertise is needed to find patterns in data and have the advantage of not being biased by personal preferences, it is an interesting task to use ML to predict the outcome of a sporting event. We have previously attempted to do so by predicting the ranking of Tour of Flanders 2018 and 2019 editions (Kholkine et al., 2020). We predicted the individual relative time of each rider and ranked the riders by the predicted relative time. Even though the study showed better performance in some of the metrics compared to fans, the approach did treat each rider independently, only was tested on one race, and did not consider how each rider in the start list compares to each other.

In this study, we look at a particular set of techniques known as Learn-to-Rank. These techniques are primarily applied in Information Retrieval to order the most relevant results to a particular query (Liu, 2009). Usually, the list of items and the relevancy weight of those items is given as input, and the output is a permutation of that list. Predicting the outcome of a road cycling race could be seen as a special case of this query-result framework, where the query is a particular race, and the results (i.e., the list to be ranked) are the individual riders. We want to understand how well this technique can correctly predict the top 10 riders of a future race. In comparison to our previous study, by pursuing a Learn-to-Rank approach, we can consider all the riders of the race when creating a ranking. To the best of our knowledge, this is the first time that such an approach is applied in predicting the outcome of a multi-contestant sports event.

Before diving into the content, it is essential to define the two major types of road cycling races: 1-day race (e.g., Tour of Flanders, Paris-Roubaix, and Liège-Bastogne-Liège) and multi-stage race (e.g., Tour Down Under, Tour de Suisse, Paris Nice, and Critrium de Dauphin Libr). As the name suggests, a 1-day race happens only on 1 day and is uninterrupted. A multi-stage race happens for several days or weeks. This is the case of the Grand Tours (Giro d'Italia, Tour de France and Vuelta a Espaa) which span over 3 weeks. In multi-stage races, the winner is the rider that can finish all the stages in the least amount of time. This study solely focuses on 1-day events with the focus on six races that are part of the “spring classics.”

We start by describing related work in section 2, whereas in section 3 we introduce the Learn-to-Rank methodology, metric, and corresponding algorithm. The proposed approach for predicting road cycling outcomes is then presented in section 4. The results are discussed in section 5, and a conclusion and possibilities for further research are given in section 6.

## 2. Related Work

Predicting sports outcomes using ML techniques has already been applied in team sports, such as rugby, ice hockey, basketball, soccer, and American football (Bunker and Susnjak, 2019) as well as in individual sports, such as hurdle races (Przednowek et al., 2014), race walking (Przednowek and Wiktorowicz, 2013), horse racing (Lessmann et al., 2009) and swimming (Xie et al., 2017). Most of the predictions in sports outcome are achieved through a multi-label classification (e.g., win, lose, draw) and some use regression to output a performance value, such as speed (e.g., Harville, 1980; Przednowek and Wiktorowicz, 2013; Przednowek et al., 2014; Spiegeleer, 2019; Kholkine et al., 2020). Besides a variety of sports, there is also a variety of ML methodologies used to predict the outcomes. Beal et al. (2020) did an extensive comparison of ML techniques for predicting outcomes of the National Football League (NFL). The authors found that the best performing techniques were Nave Bayes, AdaBoost, and Random Forest with accuracies of 68, 66, and 64%, respectively. The Nave Bayes model had a 1.7% improvement over the bookmakers. Danisik et al. (2018) predicted the outcome of soccer matches using a long short-term memory (LSTM) model with an accuracy of 52% coming close to the bookmaker's accuracy of 53%. Besides the examples presented in this study, the review by Bunker and Susnjak (2019) mentions other techniques, such as Neural Network (NN), decision trees, Support Vector Machine (SVM), boosted trees, k-Nearest Neighbors (kNN), Bayesian networks, and linear regressions (LRs). The review also points out that even though NN methods are very popular for predicting sports outcomes, they do not have the best performance in works where they are compared to other methodologies. Other popular methodologies with good results are tree-based algorithms, such as decision trees and boosting trees. In fact, Hubek et al. (2019) proposed a methodology for predicting soccer results using gradient boosting trees that won the 2017 soccer prediction challenge.

While much research in outcome prediction focuses on team sports such as soccer or, basketball, the research on techniques to predict multi-contestant sports and specifically (road) cycling is somewhat limited. Both Hobson and Goff (2017) and Spiegeleer (2019) predict how fast a particular stage in a grand tour will be. Hobson and Goff (2017) use the traditional physical model by treating each stage as a series of inclined planes and predicting the best time in each stage of Tour de France, whereas (Spiegeleer, 2019) predicts the mean velocity of each stage on all grand tours using gradient boosting. These predictions can already be helpful to understand how fast a particular race will be, but they do not give the complete picture of the race outcome. Other studies focus more on predicting the individual's rider's performance. For example, Revinskaya (2019) uses LR and NN architectures to predict the time that it takes to complete a certain segment by “casual bikers” (non-professional bikers) by using Strava^1^ data. Karetnikov (2019) predicts the Mean Maximum Power (MMP) for several time periods in races based on the training. de Leeuw et al. (2020) proposed a feature-aggregation based approach to predict the time gained or lost in a stage of a grand tour by a general classification rider compared to his direct competitors. Even though the results of these studies are promising for predicting the outcome of a race, they are done under very specific conditions and rely on sensor data that are not publicly available for all the riders of a race. This makes it impossible to predict the results of the whole peloton.

To predict the results of a race, there is a need for data regarding all riders, such as publicly available data. Spiegeleer (2019) predicted the difference between the mean velocity of the rider and the stage and head-to-head wins for all the grand tours from 2016 to 2018 using only publicly available data. For this prediction, the author used the results of the riders, weather, and the course of the race with different feature engineering processes. The author obtained a Mean Absolute Error (MAE) of 0.1576 m/s for the difference between velocities and a mean stage accuracy of 73.16% for the head-to-head wins. However, the author does not provide a prediction that ranks the riders.

As such, in our previous work, we used scrapped historical results to predict the top 10 riders of Tour of Flanders, a 1-day race. We used the gradient boosting tree library XGBoost to create a model that predicts relative finish time of the rider. The input to the model is the relative finishing time of selected races and several features built on Pro Cycling Stats (PCS) points. To obtain our top 10 prediction, we sort the riders by the predicted relative finishing time, and later we compare the results to the number of correctly predicted riders by fans in the Pro Cycling Stats game^2^. The model predicted six and four out of the top 10 riders for the 2018 and 2019 editions, respectively. In both cases, this is one rider less than the fans predicted (Kholkine et al., 2020). This model showed promising results, although several aspects could be improved in the methodology: the prediction does not take into account the whole peloton (i.e., how does each rider compare to all the other riders), and the evaluation metric does not account for which positions were predicted correctly (e.g., it is better to predict the 1st, 2nd, and 3rd rider instead of 4th, 5th, and 6th). As the goal is to predict the ranked outcome of the race, we hypothesize that a Learn-to-Rank approach is applicable to this case, which we introduce in the following section.

## 3. Learn-to-Rank

In the previous section, two ML approaches that use publicly available data to predict an outcome of a race were presented, although none of them considers the peloton as a whole. Nevertheless, several ML ranking algorithms exist that consider the complete list to be ranked (Phophalia, 2011). The most common application for the Learn-to-Rank techniques is information retrieval where retrieved documents are ranked by their relevancy to the query. Other applications include recommender systems (Kuhlman et al., 2018), construction of long-short stock portfolios (Zhang et al., 2021), and ranking the most relevant answers in an online forum (Dalip et al., 2013). In this section, we introduce the concept of Learn-to-Rank and connect it to the use case of predicting the top 10 riders in a road cycling race.

### 3.1. Introduction to Learn-to-Rank

**Figure 1:**
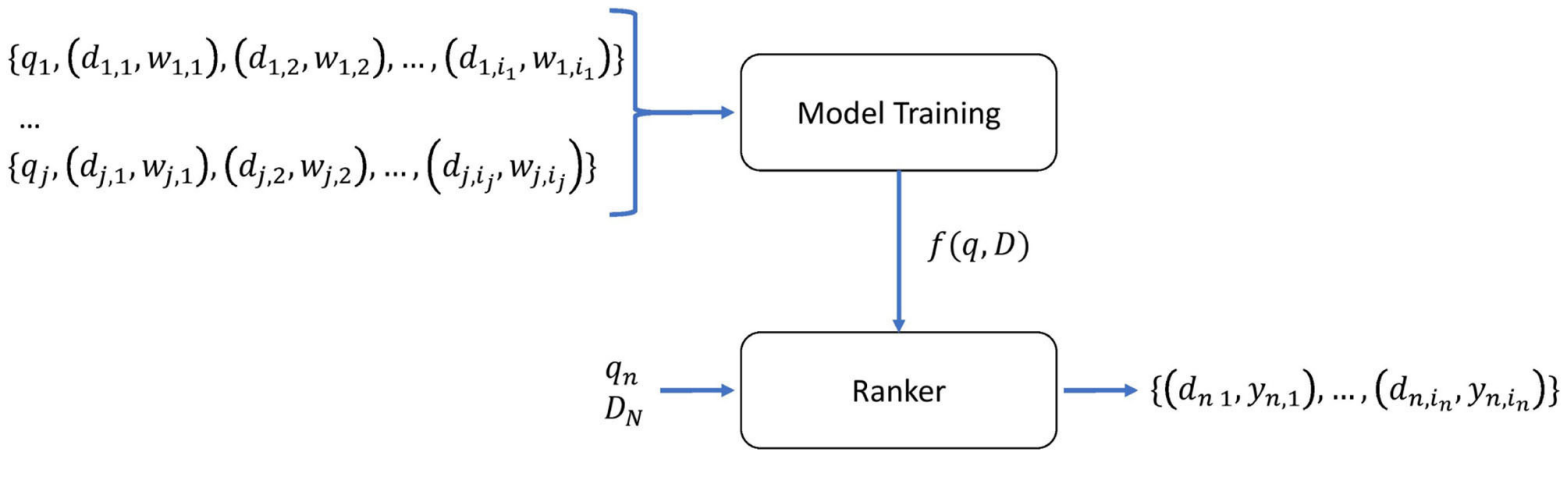
Overview of the Learn-to-Rank approach. The model is trained on *j* queries *q* to which each document *d* and a relevancy score *w* belongs. *i* represents the number of each *d,w* pairs. The trained model is a function that takes *D*_*n*_ and *q*_*n*_ which is an unseen set of documents and query and returns a *y*_*n*_ for ranking each document in *D*_*n*_.

While in traditional ML approaches the goal is to predict an unknown value from past target outputs, may it be a classification (predicting a categorical/discrete value) or regression (predicting a continuous value), the goal in Learn-to-Rank is to predict a permutation of a set of items having the most relevant items on the top of the list (Li, 2011). Figure 1 shows an overview of Learn-to-Rank. The model is trained by giving a dataset of queries and documents, represented by features extracted from them. The dataset is composed of *j* subsets of one query *q* to which each document *d* belongs and a relevancy score *w* of the document for that query. The number of document-weight *i* pairs can vary per query. The relevancy score *w* can be either binary (relevant or not relevant) or graded. The trained model is a function *f*(*q,D*) that takes as input a set of documents *D*_*n*_ and query *q*_*n*_ that it has not seen before and outputs a value *y*_*n*_ for each document by which the input documents can be ordered, providing a ranking. If each past edition of a race is grouped as a subset and we consider each rider as a document and weight a mapping to the actual result in that edition, it is possible to apply the Learn-to-Rank approach to predict a ranked top 10 riders.

### 3.2. Metrics

There are several metrics available to measure the performance of ranking algorithms. The most popular metrics include Mean Reciprocal Rank (MRR), Mean Average Precision (MAP), and Normalized Discounted Cumulative Gain (NDCG) (McFee and Lanckriet, 2010). MRR is the simplest metric, which gives a score based on where the first relevant items are predicted and is calculated by $\frac{1}{\left|Q\right|}\overset{\left|Q\right|}{\underset{i=1}{\sum }}\frac{1}{{\text{rank}}_{i}}.$ where Q is a sample of queries and rank_*i*_ is the rank of the first relevant document for the i^th^ query. MAP calculates the precision average until a certain cut-off and is calculated by $\frac{1}{Q}\overset{Q}{\underset{q=1}{\sum }}$ AveP(q), where AveP(q) is the average precision of each query q in the subset of queries Q. The main drawback of these metrics is that they are applicable in binary cases only.

For non-binary ranking, where the relevancy score can have different values, NDCG can be used (Järvelin and Kekäläinen, 2000). Similar to MAP, NDCG@k can be used to cut-off at a specific k^th^ place. The calculation of the NDCG is done in the following steps:

1. Calculate the Discounted Cumulative Gain (DCG) at k, where *w* is the graded relevance:
  $$DCG@k=\overset{k}{\underset{i=1}{\sum }}\frac{w_{i}}{\log_{2}\left(i+1\right)}$$
2. Order the ranking by *w* and use the same formula to calculate the Ideal Discounted Cumulative Gain (IDCG), which is the DCG for the ideal case scenario.
3. Calculate the NDCG score by normalizing the DCG score with the IDCG:
  $$NDCG@k=\frac{DCG@k}{IDCG@k}$$

The advantage of using NDCG as the metric is that it considers the difference in relevance between items and how they are placed in the ranking. The disadvantage is that it does neither consider misclassified results.

### 3.3. LambdaMART

The main difficulty of using Normalized Discounted Cumulative Gain (or other ranking metrics) as the objective function in ML models is that it is not differentiable nor continuous. And hence, Burges et al. (2005) proposed using stochastic gradient descent methods with the goal to minimize the number of incorrect orders among pairs of items. The methodology was implemented by the authors using an NN. Later, Burges et al. (2007) found that there is no need for the cost function for ranking, but only to estimate the gradient (lambda). Based on this, the algorithm LambdaRank was proposed. One important aspect of LambdaRank is that the gradients are scaled by the change in the NDCG score. As the previous algorithm, it was also implemented by using an NN. Wu et al. (2010) proposed implementation of LambdaRank by using Gradient Boosting Machines called LambdaMART. This adaptation increased the performance of experimental datasets. Due to success of LambdaMART's, it was implemented in the popular gradient boosting tree algorithms.

## 4. Methodology

In this study, our goal is to predict the top 10 finishers of 1-day road cycling races based on scrapped historical results and publicly available data on Pro Cycling Stats by using the Learn-to-Rank approach. More concretely, we predict the top 10 riders for 2017, 2018, and 2021 editions of E3 BinckBank Classic, Gent-Wevelgem, Tour of Flanders, Paris-Roubaix, La Flèche Wallonne, and Liège-Bastogne-Liège. We decided to predict the top 10 riders, since typically those are the riders that take most Union Cycliste Internationale (UCI) points and are the most relevant to the fans. On the other hand, the lower the ranking, the lower the reward. For example, in Tour of Flanders places between 16 and 20 receive the same number of points and no points are given below place 60.

**Figure 2:**
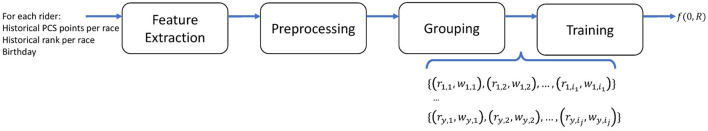
Pipeline for training a Learn-to-Rank model for 1-day road cycling races. The grouping stage outputs *y* subsets corresponding to the number of years used to train the model. Each subset contains pairs of riders *r* and an associated weight *w*. Each subset can contain a different number of riders.

To predict the top 10 riders, we first train a model for each race that we want to predict. The pipeline to train this model is represented in Figure 2 and takes as input all the historical results of each rider and birthdays of the riders. From this data, features are extracted and engineered. After all the features are constructed, it is necessary for applying the Learn-to-Rank algorithm to group them by year and give a target weight to each rider, and it is these groups that are used to train our model. We will now discuss this data gathering and preprocessing process in more detail and describe our predictive model.

### 4.1. Input Data

We used historical results and collected publicly available data from the Pro Cycling Stats website^3^ for all the riders of every edition of the race that we are predicting. This data includes the following:

- All the historical results of the rider;
- The amount of Pro Cycling Stats^4^ points attributed to the rider. We use Pro Cycling Stats points as the UCI point system changed in 2016 and does not provide consistency over the years;
- The rider's birthday.

The data algorithm was developed with the data starting from 2000 until 2017. We used the years 2018 and 2019 for testing. Later, we also predicted the top 10 riders of 1-day races in 2021 on models trained on data from 2000 until 2019. We have purposefully left out the year 2020, as it was an irregular year in the cycling calendar due to the COVID-19 pandemic. From this data, we constructed several features that are explained in the following section.

### 4.2. Feature Extraction and Engineering

Using historical data, we try to create a snapshot of the rider the day before the race and answer the following questions: How does the rider perform in similar races? What is the current shape of the rider? How well does the rider perform overall? To answer these questions, we extracted features from the historical data. The summary of these features can be found in Table 1 and each category is described in detail below.

**Table 1:** Summary of the features extracted for each of the riders of each race.

**Feature name**	**Category**	**Number** **of features**
Race results in the current year	Selected races	Varies
Race results in the previous year	Selected races	Varies
Pro Cycling Stats (PCS) points in the current year	Overall performance	1
PCS points from 1-day races in the current year	Overall performance	1
Avg. PCS points of the previous years	Overall performance	1
Avg. PCS points from 1-day races of the previous years	Overall performance	1
Avg. PCS points from selected races of the previous years	Overall Performance	1
Year-to-year difference of number of points in the last 3 years	Rider evolution	3
Linear regression slope of the points over the last 3 years	Rider evolution	1
Number of points in the 6 weeks before the races	Form	6
Best rank in the race	Best result	1
Number of years since best result	Best result	1
Age	Profile	1
Career length	Profile	1

*The Pro Cycling Stats (PCS) points represent the quality of a rider https://www.procyclingstats.com/info/point-scales*.

#### 4.2.1. Selected Races

In road cycling, different races can suit different types of riders. For example, a heavier rider might have more difficulty in mountain races but might be good at flat races. For this reason, based on our domain knowledge, we selected relevant races to the race we are predicting from the current and previous years. The criteria for selecting the race were the type of course and whether the same type of riders competes in those races. For each of the selected races, we used the ranking of the rider in that race as input. In case the rider did not complete the race (did not race at all, did not finish, did not start, or any other reason where a ranking is not attributed), we marked the value as missing. Besides the selected race, we also included the result from the previous year of the race that we are trying to predict.

#### 4.2.2. Overall Performance

Although the performance in related races can give an overview of the fitness of the rider to the race, not all the riders race the same races, and many times, due to unpredictable events, the rider might have bad results. This is why it is also important to include features that can represent the overall performance of the rider in the current year and the previous year. We measure the overall performance by the number of overall points, and 1-day race points a rider has accumulated in the current year up until the race and an average over the past years. We also include the number of points on average from selected races in the past years. The numbers of years to be considered for the average number of points in the past years was used as a hyperparameter.

#### 4.2.3. Rider Evolution Over the Years

For some of the riders, it is possible to see a trend that they have been improving over the years, and in the current year, the rider could have better results compared to the previous year. We represented this with three features:

- Difference between the number of points in year -1 and year -2;
- Difference between the number of points in year -2 and year -3;
- The slope from an LR on the number of points in the last 3 years.

**Figure 3:**
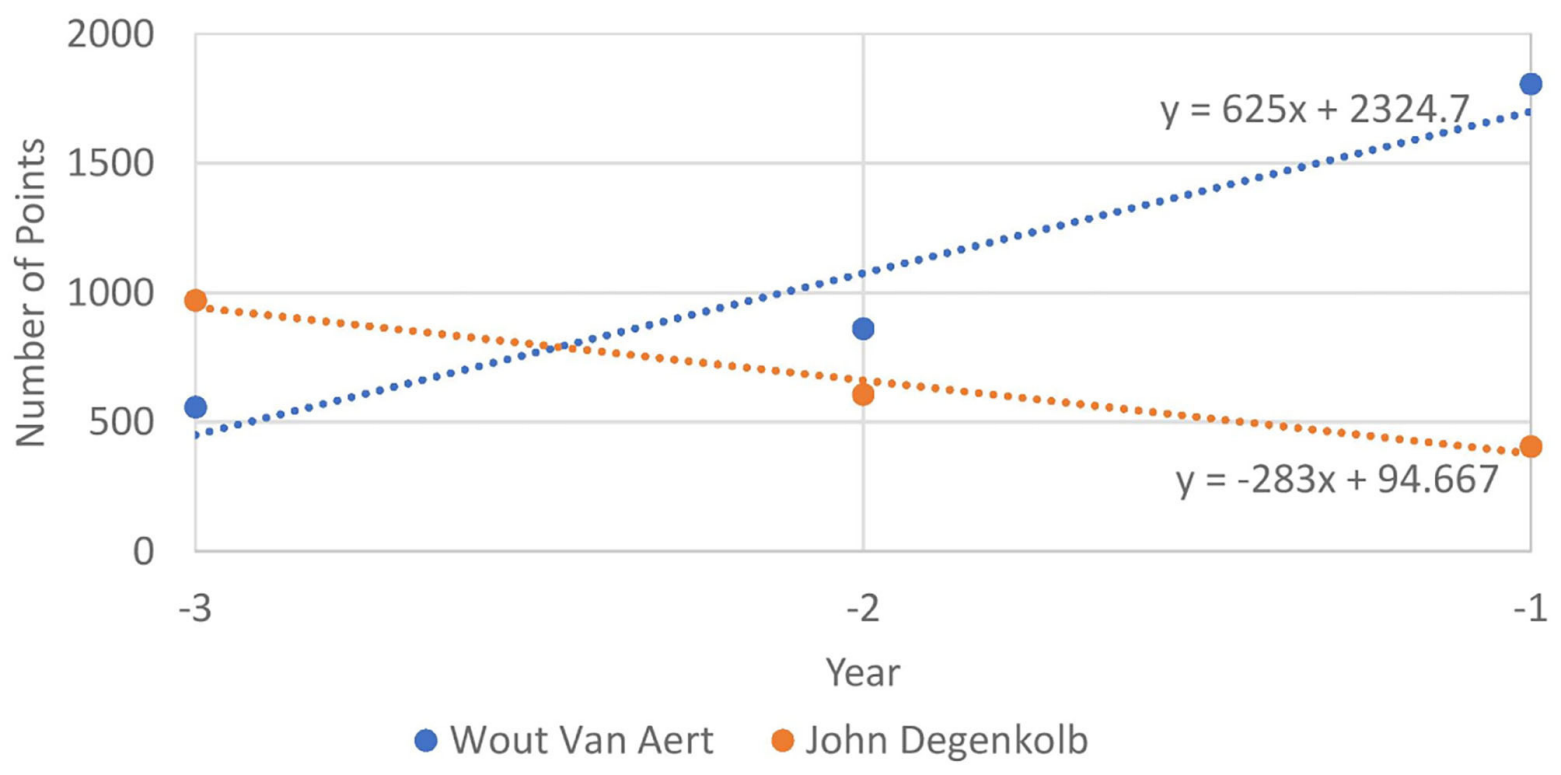
Evolution of the number of Pro Cycling Stats points that Wout van Aert and John Degenkolb won in the 3 years before 2021 (from 2018 to 2020).

We illustrate the possible importance of these features, by looking at the evolution of the riders, Wout Van Aert and John Degenkolb from 2018 to 2020 in Figure 3. We can see that Wout van Aert was on a positive trend until 2020, and, indeed, this has been confirmed by his real results in the 2021 races we predicted (win at Amstel Gold Race and Gent Wevelgem). On the other hand, John Degenkolb has a negative trend that can also be seen in his real results (no top 10 position, in the races that were predicted).

#### 4.2.4. Form

While the rider evolution and overall performance provide a good picture of the rider's form on a yearly basis and the related race provides the fitness of the rider to the race we are predicting, they might not provide enough insight into how a rider is performing right before the race. For this reason, we added *form* features. These *form* features represent how well a rider is performing 6 weeks before the start of the race. To represent the form, we sum up the number of points a rider has obtained during that period. If a rider did not participate in any race, the value was left as a missing value so that the model can interpret it as “rest days.”

#### 4.2.5. Best Result in the Race

**Figure 4:**
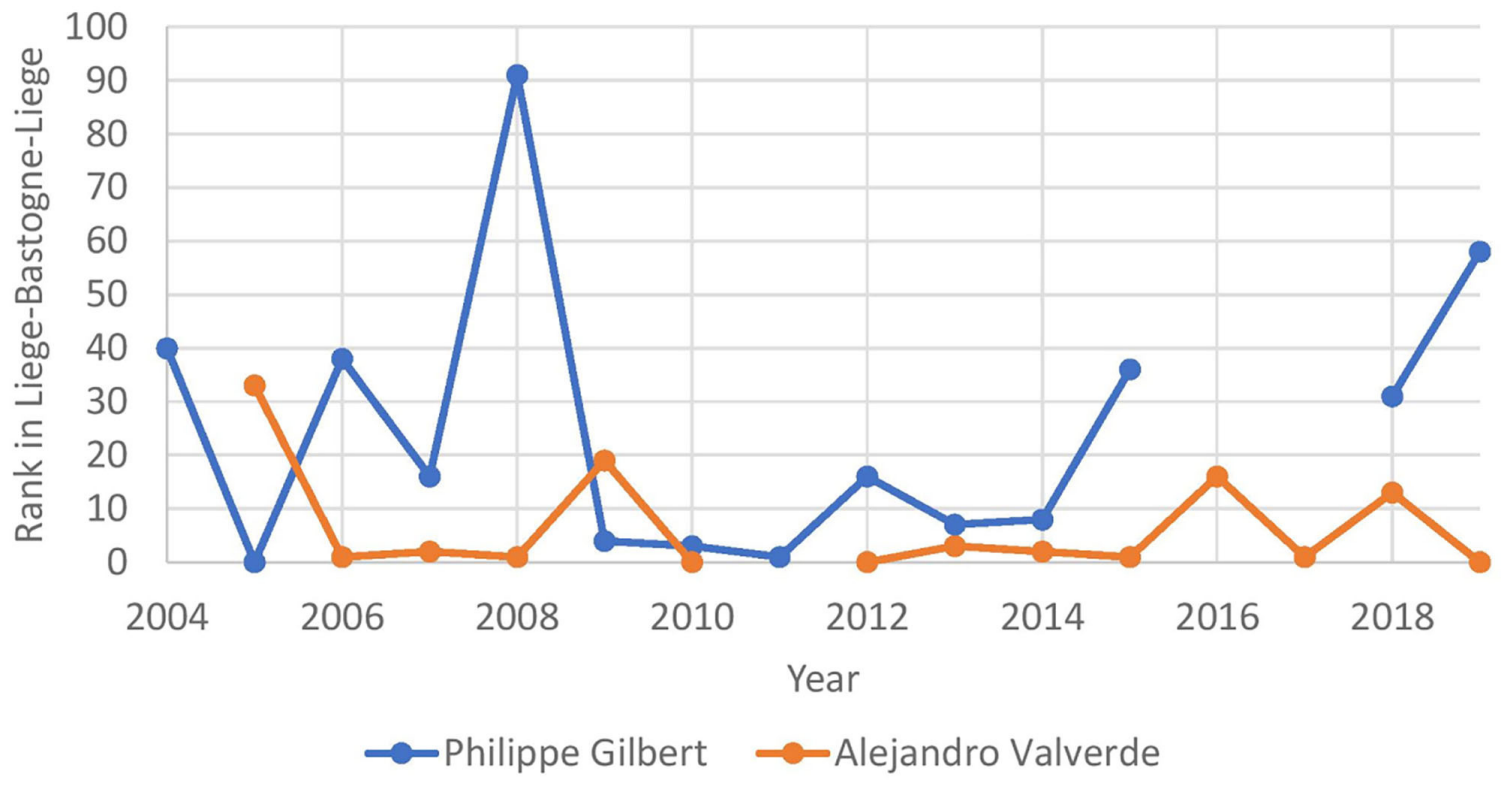
Results from Alejandro Valverde and Philipe Gilbert over the years on Liège-Bastogne-Liège.

For certain races, we noticed that some riders might have a natural decrease in the performance of a race while others might stay consistent. As an example, we take two riders with long careers: Alejandro Valverde and Philipe Gilbert. Both are riders that have raced Liège-Bastogne-Liège many times, and their rank over time is represented in Figure 4. From this figure, it is possible to see that Philipe Gilbert peaked between 2009 and 2011, while Alejandro Valverde consistently stayed in the top 20 over the years. To represent this in our data, we added a feature *best result ever* which is the best result for that rider and left it blank if the rider has never raced in that race. Next to this feature, we also added the *number of years since best result*, which represents the number of years that passed since the results were accomplished.

#### 4.2.6. Profile

**Figure 5:**
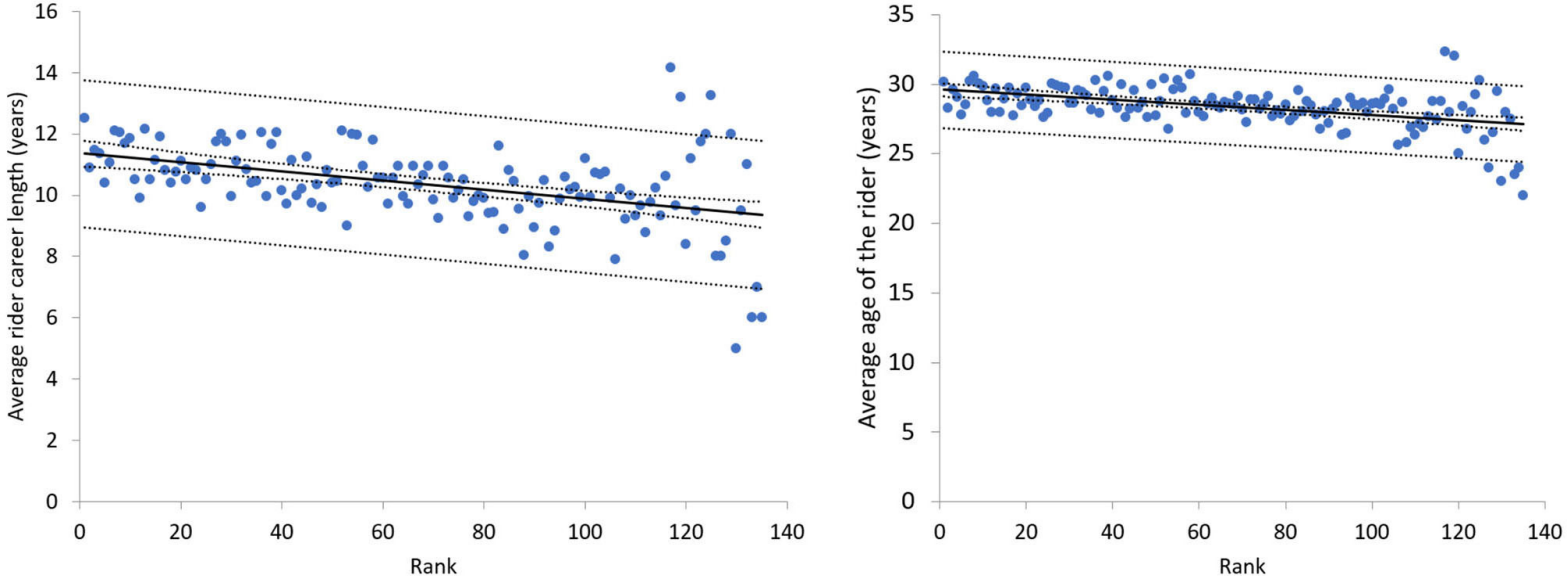
The trend of average career length (left) and age (right) relative to the rank. Each point represents the rank and the career length/age of the riders of the Tour of Flanders from 2000 to 2019.

Finally, we noticed that on average, there is a slight trend of the rank with career length of the rider and age of the rider. We exemplify those trends for all the editions of Tour of Flanders from 2000 to 2019 on Figure 5. We considered the start of the career of a rider since the first in a UCI ranked race.

### 4.3. Preprocessing

**Figure 6:**
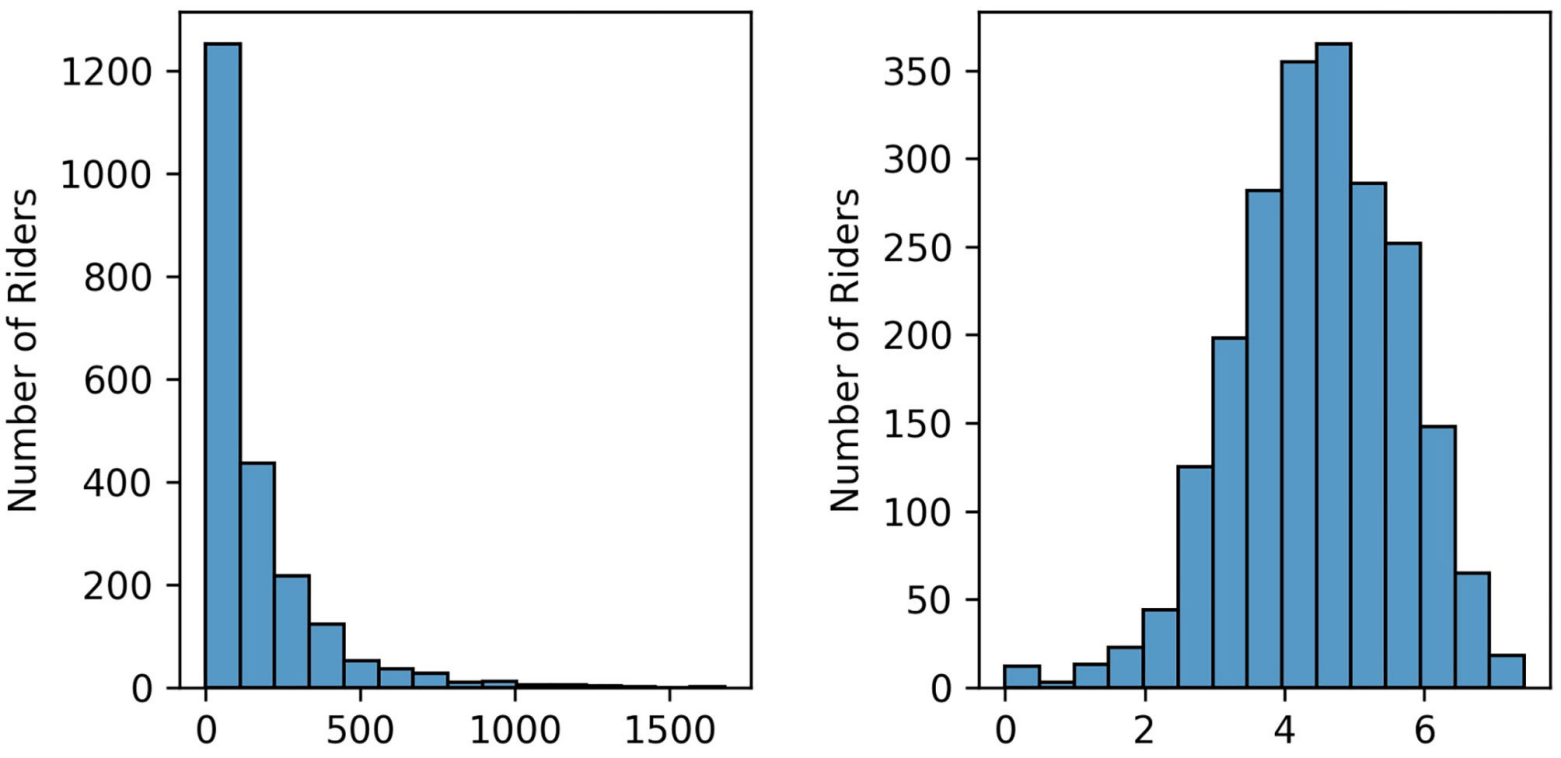
On the left: Example of distribution of Pro Cycling Stats points among riders of a 1-day race. On the right: Log transform applied to that distribution.

As the choice of algorithm (refer to the following subsection) is invariant to the scale, we did not preprocess our values. The only transformation we performed is to the *overall performance* features, as we noticed that those are heavily right-skewed. For this reason, we applied a log-transform to the whole category. A sample of the results of the transformation can be seen in Figure 6. Note that the technique used can deal with missing values, therefore no imputation operations are needed.

### 4.4. Grouping and Weighing

As mentioned previously in section 3, the Learn-to-Rank approach takes as input a number of subsets composed of one query *q* to which each document *d* belongs and a relevancy score *w* of the document for that query. We adapt to that approach as follows: we consider each rider *r* as one document and group the riders by the edition of each race having a total of *y* groups, each group representing 1 year. Since we are training one model per race, it is not needed to add any query-specific features and therefore can ignore *q*. The representation of the group can be found in Figure 2. The relevancy weights *w* are distributed as follows: 10 is given to the 1st position, 9 to the 2nd and continuing to decrement the weight until the weight is 0 at 11th place and onward.

### 4.5. Model Training

For training our model, we decided to use LambdaMART (as described in section 3) as the algorithm to build the Learn-to-Rank model. This choice is due to the fact that it uses gradient boosting trees, and as discussed in section 2, those categories of algorithms have been having much success in predicting sports outcome. LambdaMART is currently implemented in all mainstream tree boosting libraries (XGBoost Chen and Guestrin, 2016, CatBoost Prokhorenkova et al., 2018, and LightGBM Ke et al., 2017), and therefore will be less prone to errors. Moreover, Bentéjac et al. (2021) have shown that in those algorithms, all have a similar performance in classification tasks. For this study, we used XGBoost as our algorithm.

For our target and evaluation metric, we have chosen NDCG@10. As mentioned in section 2, this metric represents not only the number of correctly predicted riders but also the position of each of the predicted rider.

**Figure 7:**
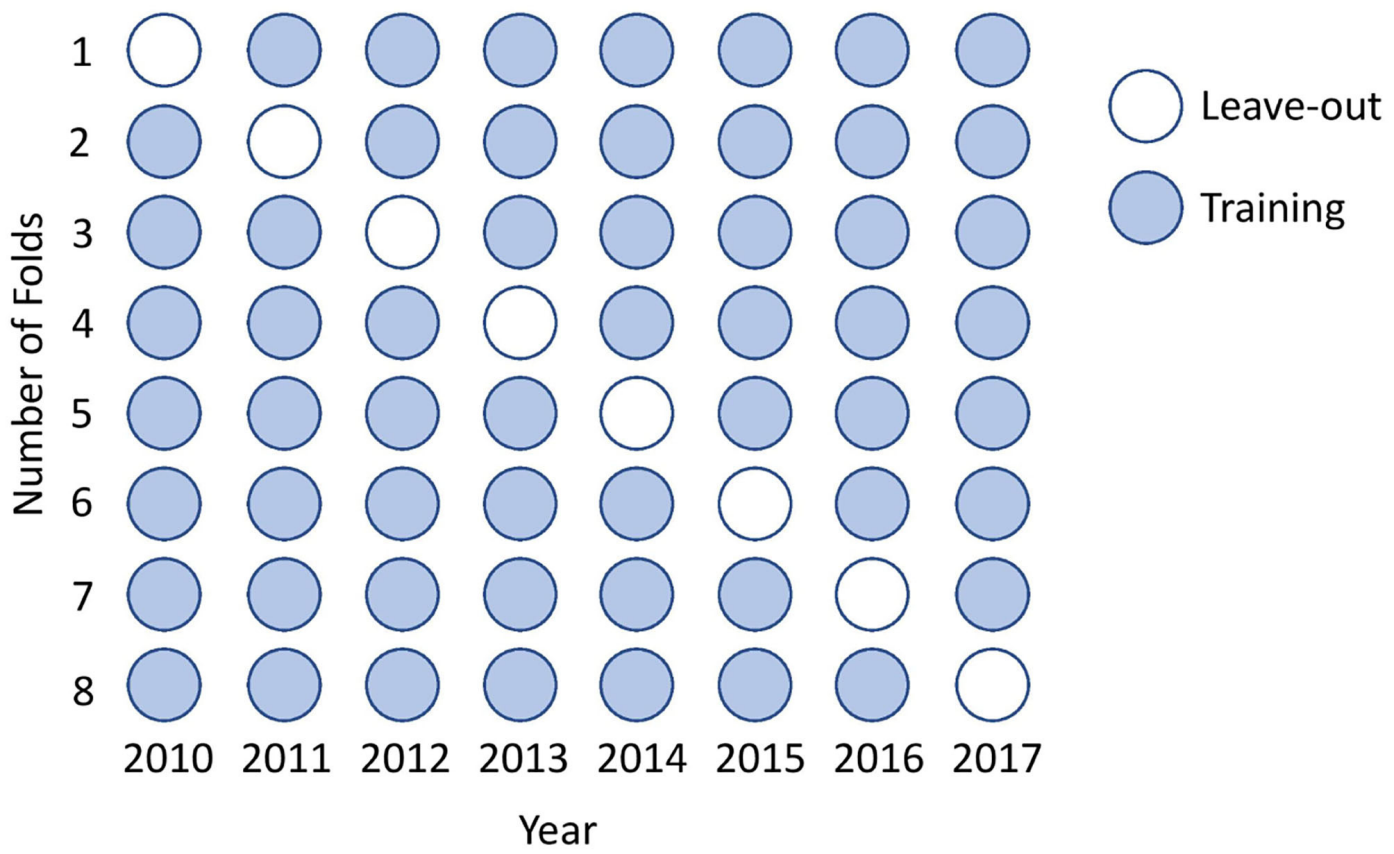
The cross-validation is performed by leaving 1-year out instead of randomly sampling the dataset.

For tuning the hyper-parameters we implemented *leave-on-year-out* cross-validation. Traditionally, a random subsample is drawn from the dataset to perform the cross-validation. In the case of Learn-to-Rank, a validation set must consist of one or more groups (i.e., result from one edition), and for that reason, we used *leave-one-year-out* as represented in Figure 7. We have also tried to generate a random subset composed of balanced riders (i.e., one rider from the first place from any edition, one from the second and so forth until a complete subset was ready), but it yielded worst results. The hyper-parameter grid is represented in Table 2. The grid refers to custom hyper-parameters which we will explain next.

**Table 2:** Hyper-parameter grid used to tune the Learn-to-Rank model.

**Parameter name**	**Type**	**Values**
Maximum depth	XGBoost	[6, 10, 15, 20, 30]
Learning rate	XGBoost	[0.001, 0.01, 0.1, 0.2, 0.5]
Subsample ratio of the training instances	XGBoost	[0.5, 0.6, 0.7, 0.8, 0.9, 1.0]
Subsample ratio of columns for each tree	XGBoost	[0.4, 0.5, 0.6, 0.7, 0.8, 0.9, 1.0]
Subsample ratio of columns for each level	XGBoost	[0.4, 0.5, 0.6, 0.7, 0.8, 0.9, 1.0]
Minimum sum of instance weight needed in a child	XGBoost	[0.5, 1.0, 2.0, 5.0, 7.0, 10.0]
Lambda	XGBoost	[0, 0.25, 0.5, 1.0]
Gamma	XGBoost	[0.1, 1.0, 5.0, 10.0]
Minimum number of boosting rounds	Pipeline	[1, 3, 5, 7, 10]
Minimum ratio of valid cross-validation folds	Pipeline	[1.00, 0.75, 0.50]
On which year to start training the model	Pipeline	[2000,2005,2010]

While training our model, we noticed that it is sensitive to overfitting due to the size of our dataset we only input at most 17 groups for training our model. To prevent overfitting, we perform early stopping on the NDCG@10 metric of the validation set at each round of cross-validation (Zhang and Yu, 2005). As one edition of a race might contain outlier results, to deal with this, we decided to create an ensemble model composed of all the *valid cross-validation folds*. We define a *valid cross-validation fold* as one that did a minimum number of boosting rounds. For an ensemble model to be considered, it must have a minimum number of *valid cross-validation folds*. The minimum number of boosting rounds and the ratio of the minimum number of *valid cross-validation folds* are tuned as hyper-parameters. All of the methodologies are shown in algorithm 1.

**Algorithm 1:**
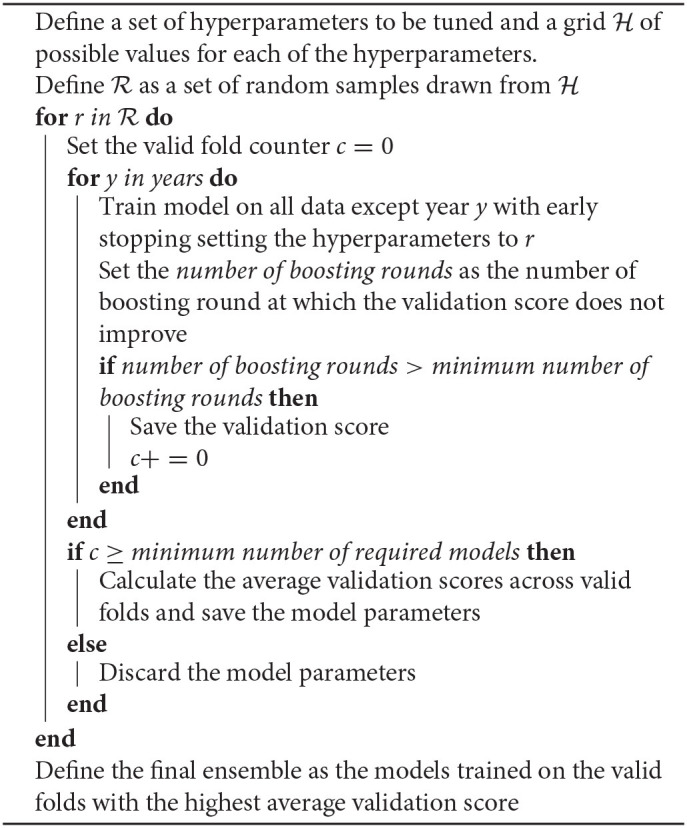
Model Training.

### 4.6. Prediction

After obtaining a trained model, it is possible to predict future races by inputting the dataset of all the riders for one edition of a certain race. To predict the ranking, we use all the models from the *valid cross-validation folds* and average their output to create the final ranking, which is shown in algorithm 2.

**Algorithm 2:**
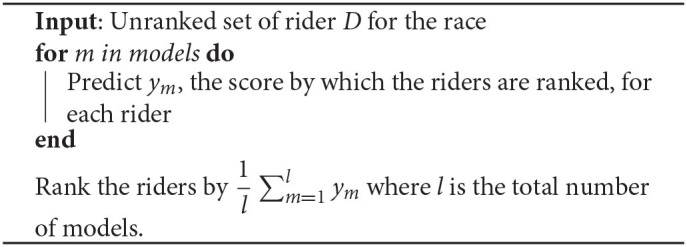
Predicting rank with trained models.

## 5. Results

In a general setting, the accuracy of a model should be assessed by comparing it to the actual results. For ranked results, this can for example be done by calculating Kendell's tau (Kendall, 1938) or Spearman's rho (Spearman, 1987) to the real result of the race. However, in many sports and specifically in road cycling, there are multiple external factors (e.g., crashes, weather, and injuries) that influence the results and cannot be captured in a data-driven approach (Phillips and Hopkins, 2020). Thus, for a fair evaluation, the performance of sports prediction algorithms is usually compared to a benchmark prediction.

A common benchmark found in sports prediction models is the bookmaker's odds, but unfortunately, to the best of our knowledge, there is no publicly available historical data for the races we considered. For this reason, we decided to compare the results to the Pro Cycling Stats game^5^. The Pro Cycling Stats game is a betting game for road cycling fans without any financial reward. Each player can pick up to five riders and distribute nine points amongst the picked riders. The number of times each rider has been chosen for each race is publicly available on the Pro Cycling Stats website. For the races predicted in this study, the number of players in the game varies between 860 (Liège-Bastogne-Liège 2018) and 1,001 (Tour of Flanders 2019). We define the fan prediction as to the list of riders sorted by the number of times a rider has been picked in the Pro Cycling Stats game. For this study, we have trained our model with the data from 2000 to 2017 and tested on the edition of 2018 and 2019 of E3 BinckBank Classic, Gent-Wevelgem, Tour of Flanders, Paris-Roubaix, La Flèche Wallonne, and Liège-Bastogne-Liège. We have also predicted those races for 2021 by using data from 2000 to 2019-, adapting our feature set by removing the races that were canceled in 2020 due to the COVID-19 pandemic.

The test result is shown in Table 3. We found that by applying this methodology, we can achieve, on average, a slightly better NDCG score compared to the fans. We also compared the number of top 10 correct predictions with the model and predict on average 5.12 riders out of the top 10 correctly. That is, on average 0.12 riders more compared to the fans. Next, we will discuss the strengths and weaknesses of this model.

**Table 3:** Fan prediction compared to the Machine Learning prediction.

**Race**	**Year**	**Fan** **Normalized Discounted Cumulative Gain (NDCG)**	**Model** **NDCG**	**Model** **correct**	**difference between** **Model and fans**
E3 SAXO BANK CLASSIC	2018	0.58	0.54	6	0
E3 SAXO BANK CLASSIC	2019	0.50	0.54	5	0
GHENT-WEVELGEM	2018	0.68	0.62	5	−1
GHENT-WEVELGEM	2019	0.23	0.32	3	0
TOUR OF FLANDERS	2018	0.62	0.67	6	−1
TOUR OF FLANDERS	2019	0.27	0.21	4	−1
PARIS-ROUBAIX	2018	0.77	0.74	6	0
PARIS-ROUBAIX	2019	0.35	0.44	4	0
LA FLÈCHE WALLONNE	2018	0.57	0.60	5	2
LA FLÈCHE WALLONNE	2019	0.55	0.61	5	1
LIÈGE-BASTOGNE-LIÈGE	2018	0.28	0.38	5	1
LIÈGE-BASTOGNE-LIÈGE	2019	0.43	0.31	3	−1
E3 SAXO BANK CLASSIC	2021	0.32	0.37	3	−1
GHENT-WEVELGEM	2021	0.41	0.63	5	3
TOUR OF FLANDERS	2021	0.69	0.69	7	0
LA FLÈCHE WALLONNE	2021	0.84	0.76	6	−1
LIÈGE-BASTOGNE-LIÈGE	2021	0.69	0.81	8	1
AVERAGE:		0.52	0.55	5.12	0.12

*The Normalized Discounted Cumulative Gain (NDCG) of the fans and the model are compared for each race and year. Also, the number of riders that finished in the top 10 and were also predicted to finish in the top 10 by the model is shown with it's difference to the fan prediction. The year 2021 results are shown separately due to the changes in the feature set caused by the COVID-19 pandemic*.

**Figure 8:**
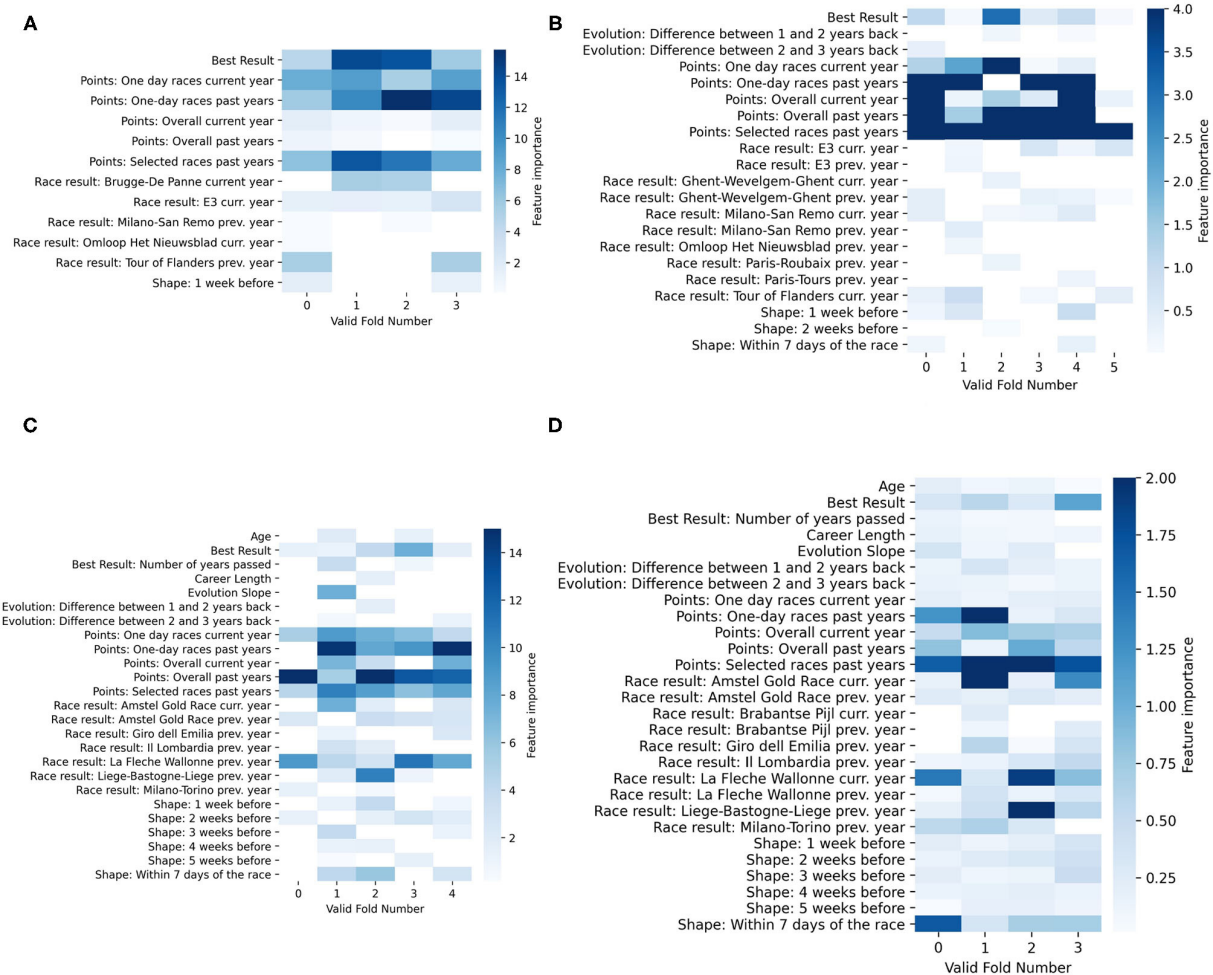
Feature importance for each of the valid cross-validation fold of the model of Tour of Flanders **(A)**, Paris-Roubaix **(B)**, La Flèche Wallonne **(C)**, and Liège-Bastogne-Liège **(D)**. The feature importance is determined by the average gain of the splits of the trees that use that feature. The higher the value, the more important is the feature. In **(B–D)**, the values have been clipped for visualization purposes.

To understand how the different race models work, we analyzed the feature importance by determining the average gain of the splits of the trees that use a certain feature. As our model is composed of multiple models, we decided to illustrate the feature importance of each of the models on a heatmap where each column represents a *valid cross-validation fold*. As examples, the feature importance of the models for Tour of Flanders, Paris-Roubaix, La Flche Wallone, and Ligie-Bastogne-Ligie is shown in Figure 8. The features presented in the heatmaps are only the ones that XGBoost selected, and for some of the figures, we clipped the values for visualization purposes.

When comparing the feature importance, it is already noticeable that depending on the race, the number of features and the feature importance can vary significantly. For example, the feature importance of Tour of Flanders model relies heavily on the best historical result achieved in Tour of Flanders and the overall performance (refer to Figure 8A), whereas the Liège-Bastogne-Liège model relies less on the overall performance feature and much more on the results from La Flèche Wallonne (refer to Figure 8C). However, all the models give importance to at least one feature related to the overall performance. This is because the model needs to predict all riders in the peloton, and having many riders with a low number of average points (as shown in Figure 6), will have a low chance of finishing in the top 10. On the other hand, it also points to the fact that the model might be looking for riders that have consistency in the results.

Riders that were predicted by our model and not by the fans, were usually the riders that are not clear favorites in the race but have been having an overall good season and some good results in the selected races. Such examples are Romain Bardet and Michael Matthews, who were predicted by the model in La Flèche Wallonne 2018. A more recent example is Ghent-Wevelgem, where Giacomo Nizzolo was predicted very low by the riders, but in fact, his Pro Cycling Stats points have been slowly increasing over the years, and he did have good results in several selected races.

On the other hand, by giving priority to consistency, the model can also miss some predictions that are obvious to fans. For example, riders that are recovering, or riders that do other types of cycling sports, as it was in the case of Mathieu van der Poel in 2019 riders that also do other cycling sports, as is the case of Mathieu van der Poel. The model predicted 46th place, and he finished 4th with a fan prediction of 5th.

Another type of consistency the model looks for is results in the previous races. This can, for example, be seen in Figures 8A,D, where there is clear importance of results in the past races. This can affect the model negatively in comparison to the fans. For example, Jasper Stuyven was predicted 10th by the fans and 14th by the algorithm while finishing 7th on the Tour of France 2018. During that year, Stuyven indeed did show potential in having a good result on Tour of Flanders. However, because the model gives importance to the best result in Tour of Flanders of the previous years and the number of points in selected races, he was not predicted in the top 10. Another example is Paris-Roubaix 2019, where Nils Politt finished 4th, and while the fans predicted 8th place, the model predicted him to finish 20th. Looking at Figure 8B, we observe that this is a consequence of the fact that the model relies heavily on points of riders in the previous years, and in 2019, Nils Politt conquered almost double the points that he had in the previous year.

Despite the shortcomings, the model does make a prediction on pair with the fan prediction and can provide a useful insight that is truly data-driven. We also believe that these insights can make the expert prediction much stronger. In the following section, we discuss some possible solutions to the shortcomings of the model.

## 6. Conclusions

We presented a Learn-to-Rank approach for predicting the ranking of the top 10 riders in a road cycling race. The approach uses an implementation of XGBoost of LambdaMART to learn a ranking predictor and uses only publicly available data. We proposed using an ensemble of models trained using k-folds cross-validation with early stopping and a mechanism that prevents using the model that did not generalize into one or more validation sets. After testing the in 2018, 2019, and 2021 editions of E3 BinckBank Classic, Gent-Wevelgem, Tour of Flanders, Paris-Roubaix, La Flèche Wallonne and Liège-Bastogne-Liège, we obtained on average a slightly better results than the fans and predicting several riders in the top 10 that the fans did not.

To understand the performance of our model, we analyzed the predictions and the feature importance. We noticed that the model has a preference for consistency of a rider either in his career (number of points) or related races and does not do well with very fast-rising stars. Most of those misses of the models are related to the fact that the model is looking at the consistency of each rider and therefore rely heavily on the numbers of accumulated points or particular races in the season, where missing one will impact the prediction of the next race. Even though we introduced features that improved this (such as the form), the model does not seem to learn a lot from them. One of the reasons that this might happen is due to the small dataset per race.

The issues mentioned can be addressed in future study by creating features related to the skills of the rider or by using a graph-based ranking or elaborating an approach to generalize the algorithm through query-specific (i.e., race-specific) features. The latter would eliminate the need to train a separate model for each race and allow the use of a single large dataset for different races. There is also room for improvement in the normalization of features that use the number of points, as those are influenced directly by the number of races done. Other future works include course, weather, and other edition-specific features, as currently, our approach assumes certain stability over the years. Finally, this study is limited to the six spring classic races presented here, and it would be interesting to apply our approach to other races. For example, an interesting avenue for future research is a generalization to multi-day races (i.e., Tour de France).

Nevertheless, this study shows the potential of using ML techniques and specifically Learn-to-Rank to predict the relative performance of riders in races. Developing further such an algorithm can help coaches understand who the main adversaries are in a race. It can also help spot the important factors for winning a specific race and adapt strategy of the team if certain riders display patterns of possibly winning a future race. Another possible direction to explore is the usage of recommendation systems that have been linked with Learn-to-Rank approaches to create recommendations of riders for a specific race or vice-versa. Finally, a feature importance analysis can better understand which race influences which other race in the future.

Besides coaches, these predictions can also be interesting for sports journalists and fans by creating data-driven predictions and bringing different insights. To showcase this application, we created the website Who Will Win the Race^6^ which, based on the models described in this work, shows the evolution of the prediction of the rider for a certain race during the season.

To conclude, we see this tool as helpful for coaches to find patterns in data and support their strategical decisions. On the other hand, it can be useful for commentators and broadcasters to give an overview of what story the data tells.

## Data Availability Statement

Publicly available datasets were analyzed in this study. The data have been obtained from https://firstcycling.com and https://procyclingstats.com.

## Ethics Statement

Written informed consent was not obtained from the individual(s) for the publication of any potentially identifiable images or data included in this article.

## Author Contributions

LK contributed to the data retrieval, coding and writing of the manuscript. TS contributed to the code review. TS, A-WDL, TDS, and TV contributed to manuscript revision, and have read and approved the submitted version. All authors contributed to the conception and design of the work.

## Funding

This study was partly funded by the DAIQUIRI project, co-funded by imec, a research institute founded by the Flemish Government. Project partners are Ghent University, InTheRace, Arinti, Cronos, VideoHouse, NEP Belgium, and VRT, with project support from VLAIO under grant no. HBC.2019.0053.

## Conflict of Interest

The authors declare that the research was conducted in the absence of any commercial or financial relationships that could be construed as a potential conflict of interest.

## Publisher's Note

All claims expressed in this article are solely those of the authors and do not necessarily represent those of their affiliated organizations, or those of the publisher, the editors and the reviewers. Any product that may be evaluated in this article, or claim that may be made by its manufacturer, is not guaranteed or endorsed by the publisher.

## Footnotes

^1^ http://www.strava.com/

^2^ https://www.procyclingstats.com/info/pcs-game

^3^ https://www.procyclingstats.com/

^4^ https://www.procyclingstats.com/info/point-scales

^5^ https://www.procyclingstats.com/info/pcs-game

^6^ https://whowillwintherace.com/en/
